# Genetic variants in ERBB4 is associated with chronic hepatitis B virus infection

**DOI:** 10.18632/oncotarget.6650

**Published:** 2015-12-18

**Authors:** Yao Liu, Qun Zhou, Xiao-Shun He, Li-Ming Song, Lin Chen, Wei-Juan Jiao, Tong Shen, Su Yao, Hua Wu, Zhi-Bin Hu, Tian-Ming Gao, Jian-Ming Li

**Affiliations:** ^1^ Department of Pathology, Medical College of Soochow University, Suzhou 215123, People's Republic of China; ^2^ Department of Epidemiology, School of Public Health, Nanjing Medical University, Nanjing 211166, People's Republic of China; ^3^ Department of Pathology, Nanfang Hospital, Southern Medical University, Guangzhou 510515, People's Republic of China; ^4^ Department of Neurobiology, School of Basic Medical Sciences, Southern Medical University, Guangzhou 510515, People's Republic of China

**Keywords:** ERBB4, hepatitis B virus, polymorphism, inflammation

## Abstract

**Background:**

The role of ERBB4 in liver disease has seldom been reported. This study aims to find genetic markers at *ERBB4* for chronic hepatitis B virus (HBV) infection and determine the role of ERBB4 in liver injury.

**Methods:**

We selected and genotyped three single nucleotide polymorphisms and one insertion/deletion (Ins/Del) at the 5′ and 3′ untranslated region (UTR) of *ERBB4* in a case-control study including 1344 pairs of HBV carriers and HBV natural clearance subjects. The luciferase reporter system was applied to study the regulative role of Ins/Del on ERBB4. Further, ERBB4 knockout mice were used to study the role of ERBB4 in liver injury. Proteomic quantification was performed by HPLC-MS/MS analysis to identify liver protein profile change between liver-specific ERBB4 knockout and control mice.

**Results:**

rs6147150 Ins/Del and rs1836724 T>C at the 3′ UTR of *ERBB4* were associated with reduced risk of chronic HBV infection (*P* = 0.002 and 0.004, respectively). Besides, the 12bp deletion at the 3′ UTR increased ERBB4 expression due to lacking let-7c binding site. In addition, loss of ERBB4 led to more severe acute or chronic inflammation in mouse liver injury models. Further, quantitative proteomic analysis and data from the cancer genome atlas revealed that ACLY, an enzyme key for *de novo* lipogenesis, was negatively correlated with ERBB4.

**Conclusions:**

ERBB4 plays protective role from liver injury and its 3′UTR genetic variants could be genetic markers for chronic HBV infection.

## INTRODUCTION

Hepatitis B virus (HBV) infection is a major disease burden in the world, especially in China [1]. Chronic HBV infection, as well as other liver injury factors, including alcohol consumption and aflatoxin contamination, are risk factors for hepatocellular carcinoma (HCC) [2–4]. However, the molecular basis for liver injury is still unclear. Previously, we have found new loci associated with chronic HBV infection in genome-wide association study (GWAS)[5]. However, loci not confirmed in our previous study cannot be simply discarded. Genetic variants with nominal association (1× 10^−4^ < *P* < 0.05) may be candidate genes, but they need more cautious validation both in population study and in functional validation. In addition, whether the hepatitis-related loci associated with multiple liver injury factors needs to be clarified.

ERBB family, including EGFR, HER2, ERBB3 and ERBB4, plays vital role in physiological process, as well as in many disorders [6]. Loss of signaling by any ERBB member results in embryonic lethality in mice [6]. Insufficient ERBB signaling in humans is associated with many diseases such as multiple sclerosis and Alzheimer's Disease [7]. Excessive ERBB signaling is associated with the development of a wide variety of solid tumor [8–10]. For example, EGFR, also named ERBB1, is over-expressed in many tumors and initiates the key step for malignant transformation [8]. HER2, also named ERBB2, is now considered as a key oncogene in breast cancer and colorectal cancer [8]. ERBB3 has also been demonstrated to be involved in a variety of cancer [11–13]. Compared with other ERBB family members, the role of ERBB4 is controversial [14–21]. On one hand, ERBB4 promotes growth of human breast cancer cells [22] and promotes metastasis in Ewing sarcoma [23]. On the other hand, activation of ERBB4 leads to cell cycle arrest, differentiation and apoptosis of breast cancer cells [16, 24]. Besides, the role of ERBB4 in liver inflammation has never been studied. Previously, Ding *et al*. reported that ERBB4 is one of those recurrent HBV target genes when the viral DNA integrate into host chromosomes [25]. All of the above have provoked our interest to study the role of ERBB4 in liver inflammation.

Genetic variation studies provide us an initial way to discover the role of ERBB4 in chronic HBV infection. In this study, we hypothesized that single nucleotide polymorphisms (SNPs) in *ERBB4* may contribute to risk of chronic HBV infection. To test our hypothesis, we conducted a case-control study including 1344 HBV carriers and 1344 subjects with HBV natural clearance to assess the associations between genetic variants at *ERBB4* and the susceptibility to chronic HBV infection. Then we evaluated the association of genetic variants at *ERBB4* with chronic HBV infection based on our existing genome-wide association study (GWAS) data set (937 cases and 951 controls). We further applied *in vitro* and *in vivo* study to assess the role of the significant variations in ERBB4 and the role of ERBB4 in liver injury.

## RESULTS

### Two genetic variants at the 3′ UTR of *ERBB4* were protective markers for chronic HBV infection

The demographic characteristics of the 1344 HBV persistent carriers and 1344 persons with HBV natural clearance have been summarized previously [26]. Briefly, no significant difference was detected in the distribution of age and gender between the two groups. Smoking and drinking rates were also similar between the two groups.

**Figure 1:**
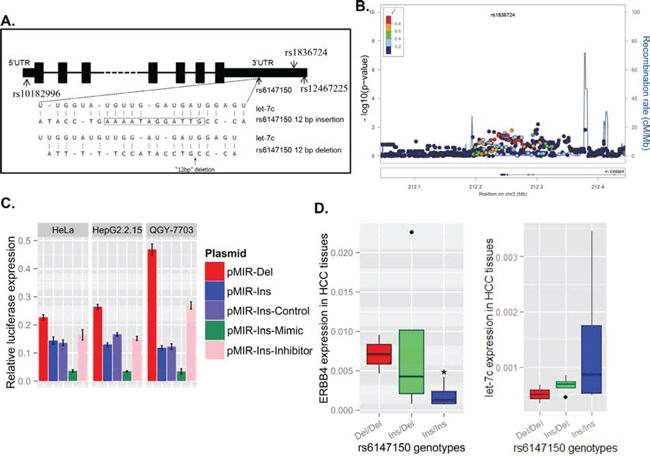
Genetic variants at the 3′UTR of ERBB4 are associated with decreased risk of hepatitis B virus (HBV) infection by regulating ERBB4 expression **(A)** The location of the four potential functional SNPs are shown in the *ERBB4* gene schematic map. Bioinformatics (MiRanda) predicted that the 12bp deletion at the 3′UTR of *ERBB4* may interrupt the binding of miRNA let-7c. **(B)** A series of significant signals around rs1836724 was observed in the regional plot of the association between genetic variants and chronic HBV infection. The linkage disequilibrium values (r^2^) for the other SNPs are indicated by the heat scale. The direction of ERBB4 transcripts shown in arrows. **(C)** The sequence containing the 12bp deletion range of upstream 1000bp to downstream 1000bp was cloned to the reporter system (pMIR-Del), and the sequence containing the 12bp were also cloned and named pMIR-Ins. The pMIR-Del plasmid demonstrated lower luciferase expression levels than pMIR-Ins in HeLa, HepG2.2.15 and QGY7703 cell lines. let-7c mimic or inhibitor cotransfected with pMIR-Ins may decrease or increase the luciferase expression level, respectively. **(D)** HCC patients with different genotypes of the 12 bp variants exhibited different ERBB4 mRNA and let-7c level in HCC tissues (n=10).

The genotype distribution of SNPs between HBV carriers and HBV natural clearance subjects was shown in Table 1. Our results showed that both rs6147150 Ins>Del and rs1836724 T>C at 3′UTR were associated with reduced risk for chronic HBV infection (rs6147150 Ins>Del, *P* = 0.002; rs1836724 T>C, *P* = 0.004). Interestingly, in our previous GWAS data [5], we observed a series of significant signals around rs1836724 in the regional plot (Figure 1B, Supplemental Table 4), and all these strongly linked loci exhibited protective effect from chronic HBV infection, suggesting the association is convincing. Based on our data, we also found strong linkage disequilibrium between rs6147150 and rs1836724 (D' = 0.989, r^2^ = 0.957), showing the person tends to carry both rs1836724 C and rs6147150 Del simultaneously. Thus, we focused on the 12 bps Ins>Del in our following study.

**Table 1:** Genotype frequencies of SNPs and susceptibility to chronic HBV infection

Genotype	HBV carriers	HBV natural clearance subjects	OR(95%CI)	*P*
rs10182996	n (%)	n (%)		
TT	1125 (84.8)	1098 (83.4)	1	
1CT	193 (14.6)	203 (15.4)	0.93 (0.75-1.15)	
CC	8 (0.6)	16 (1.2)	0.48 (0.21-1.14)	
CT/CC	201 (15.2)	219 (16.6)	0.90 (0.73-1.10)	0.302
Additive			0.88 (0.72-1.06)	0.177
rs6147150	n (%)	n (%)		
II	778 (58.2)	707 (53.1)	1	
ID	484 (36.2)	519 (39.0)	0.85 (0.72-0.99)	
DD	75 (5.6)	106 (8.0)	0.64 (0.47-0.88)	
ID/DD	559 (41.8)	625 (46.9)	**0.81 (0.70-0.95)**	**0.007**
Additive			**0.82 (0.73-0.93)**	**0.002**
rs1836724	n (%)	n (%)		
TT	772 (57.7)	708 (53.1)	1	
CT	485 (36.2)	513 (38.5)	0.87 (0.74-1.02)	
CC	81 (6.1)	112 (8.4)	0.66 (0.49-0.90)	
CT/CC	566 (42.3)	625 (46.9)	**0.83 (0.71-0.97)**	**0.017**
Additive			**0.84 (0.74-0.95)**	**0.004**
rs12467225	n (%)	n (%)		
CC	601 (45.1)	604 (45.2)	1	
CT	607 (45.5)	590 (44.2)	1.04 (0.88-1.22)	
TT	126 (9.4)	142 (10.6)	0.89 (0.68-1.16)	
CT/TT	733 (54.9)	732 (54.8)	1.01 (0.87-1.18)	0.910
Additive			0.98 (0.87-1.10)	0.704

NOTE: Logistic regression analyses adjusted for age, gender, smoking status and drinking status. Abbreviations. II: Ins/Ins. ID: Ins/Del. DD: Del/Del.

Compared with the HBV carriers with wild genotype of rs6147150 Ins/Ins, those with Ins/Del and Del/Del genotypes had a decreased risk for chronic HBV infection with adjusted ORs of 0.85 (95% CI = 0.72-0.99) and 0.64 (95% CI = 0.47-0.88). The variant genotypes ID/DD of rs6147150 significantly decreased chronic HBV infection risk (adjusted OR = 0.83, 95% CI = 0.71-0.97, *P* = 0.017 in dominant model; adjusted OR = 0.84, 95% CI = 0.74-0.95, *P* = 0.004 in additive model).

The association between rs6147150 and the susceptibility of chronic HBV infection was also evaluated by stratifying age, gender, drinking and smoking status (Supplemental Table 5). However, no significant heterogeneity was detected between subgroups, implying independent genetic effect of rs6147150.

### The 12bp deletion at the 3′ UTR impairs ERBB4 expression by inhibiting miRNA binding

The 12 bp deletion at the 3′UTR may increase ERBB4 expression by suppressing let-7c binding, which is predicted by miRanda. To test the effect of the 12bp deletion, the 3′ UTR of *ERBB4* with or without the 12 bp deletion were cloned to a luciferase reporter system to evaluate gene regulation. Interestingly, the 12bp deletion at the 3′ UTR of *ERBB4* (pMIR-Del) greatly increased the luciferase expression compared with that with 12 bp insertion (pMIR-Ins). In addition, transfection of let-7c mimic could inhibit luciferase expression level while let-7c inhibitor could increase luciferase expression when cotransfected with pMIR-Ins (Figure 1C). In human liver tumor tissues obtained from surgery, the mRNA levels of ERBB4 with genotypes Del/Del and Ins/Del of rs6147150 were elevated compared with that with Ins/Ins of rs6147150, while the expression level of let-7c was in the reverse trend (Figure 1D), supporting the regulatory role of a 12bp Ins/Del at the 3′ UTR of *ERBB4*.

### ERBB4 may play protective role in chronic HBV infection

**Figure 2:**
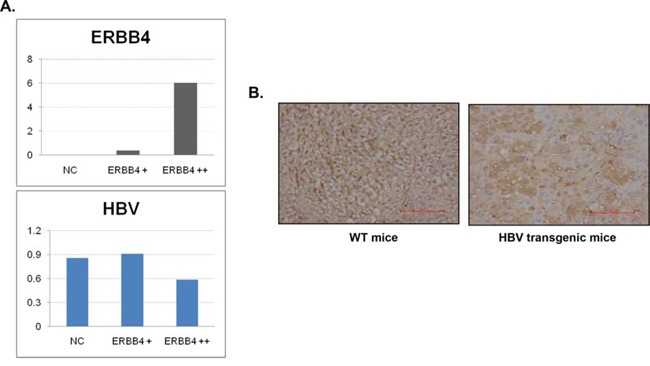
ERBB4 may play protective role in chronic HBV infection (**A**) 48 h after ERBB4 over-expression, the expression of HBV mRNA significantly decreased (P<0.001). (**B**) Expression intensity and location of ERBB4 were shown in wild type mice (WT) and HBV transgenic mice (HBV) performed by immunohistochemistry.

We transfected ERBB4 in HepG2.2.15, a cell line with HBV stable expression, the expression level of HBV decreased, suggesting ERBB4 promote HBV clearance (Figure 2A). In addition, in normal mice livers, ERBB4 was expressed in the cell membrane. However, in HBV transgenic mice, the expression level of ERBB4 decreased and the location shifted to cytoplasm (Figure 2B), suggesting the impairment of ERBB4 in HBV infection.

### Loss of ERBB4 leads to more severe acute liver inflammation in mice models

**Figure 3:**
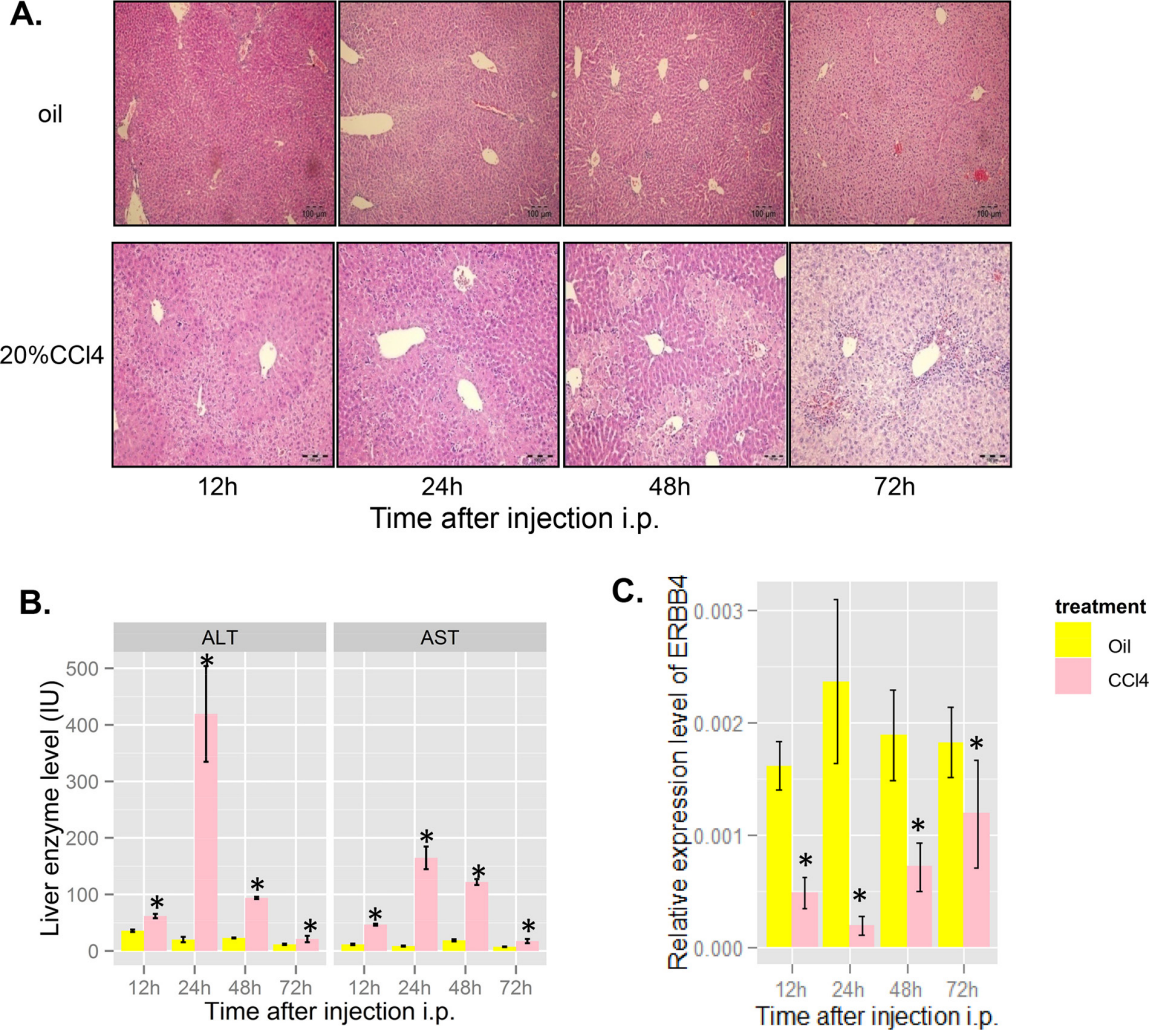
ERBB4 expression decreased initially and recovered after liver injury induced by acute carbon tetrachloride (CCl4) in mice **(A)** Hematoxylin and eosin staining (H&E) of liver sections in mice after acute CCl_4_ administration. **(B)** Serum levels of alanine aminotransferase (ALT) and aspartate aminotransferase (AST) in mice after oil or CCl_4_ injection, respectively. **(C)** ERBB4 mRNA expression levels in mice liver after oil or CCl_4_ injection, respectively.

Liver injury induced by CCl_4_, a classic model of chemical liver injury, was established in mice to study the role of ERBB4 in liver injury. H&E of liver sections showed that acute CCl_4_ administration caused massive centrilobular injury, which was still observed 72 hours after treatment (Figure 3A). Serum ALT and AST were significantly higher in CCl_4_ treated mice than those in controls (Figure 3B). Interestingly, we found that ERBB4 expression decreased significantly and restored in some degree during the progression of liver injury induced by CCl_4_ (Figure 3C).

**Figure 4:**
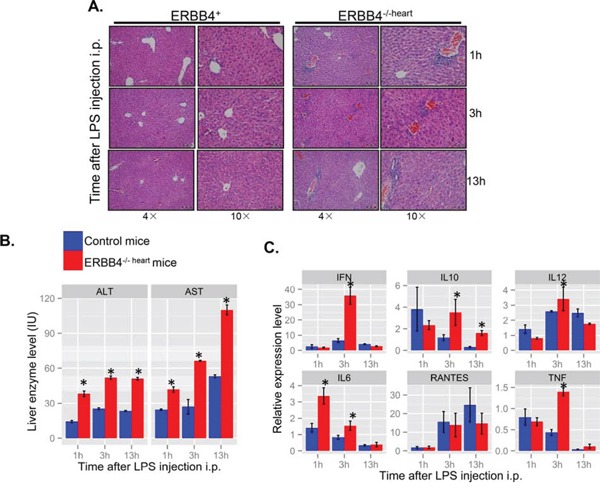
ERBB4 knockout mice exhibited more lymphocytes infiltration in lipopolysaccharides (LPS)-induced acute liver injury **(A)** Hematoxylin and eosin staining (H&E) of liver sections at different time points after lipopolysaccharides (LPS) intraperitoneal (i.p.) injection in ERBB4^+/+^ and ERBB4^−/−heart^ mice. **(B)** Serum ALT and AST levels at different time points after LPS i.p. injection. **(C)** Inflammatory cytokines change in the liver at different time points following LPS i.p. injection.

To further define the role of ERBB4 in hepatic inflammatory responses, we assessed induction of liver damage by LPS challenge in ERBB4^−/−heart^ mice. Intraperitoneal LPS injection elicited more severe liver damage response in ERBB4^−/−heart^ mice than in controls. H&E of liver sections also revealed increased inflammatory cell infiltration into hepatic parenchyma (Figure 4A). Also, liver cells damage was more severe in ERBB4^−/−heart^ mice compared with those in ERBB4^+/+^ mice, as indicated by higher serum ALT and AST levels (Figure 4B). We measured local levels of inflammatory cytokines in the liver tissues at different time points following LPS injection, and detected higher levels of IFN, IL12, IL-6 and TNF in ERBB4^−/−heart^ mice than in control mice three hours after LPS injection (Figure 4C), suggesting an enhanced inflammatory reaction in ERBB4^−/−heart^ animals. These results from *in vivo* study suggested protective role of ERBB4 in acute liver injury.

### Loss of ERBB4 leads to more severe alternative inflammation after long-term liver injury in mice models

**Figure 5:**
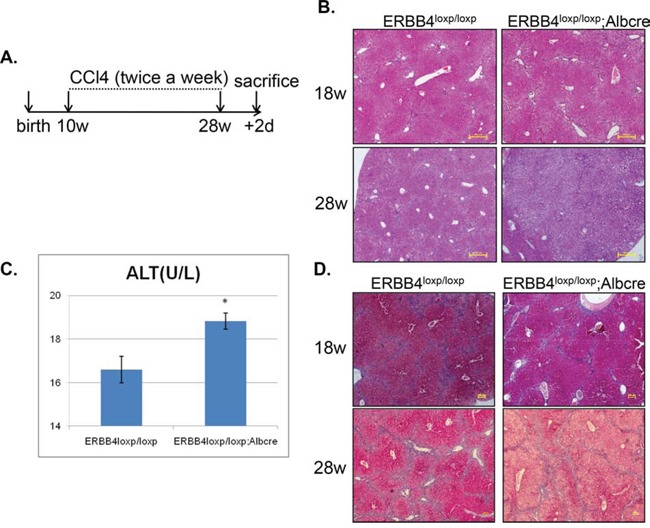
ERBB4 liver-specific knockout mice exhibited severe alterative inflammation after long term carbon tetrachloride (CCl_4_) administration **(A)** Schematic depiction of experimental design. Mice were injected with CCl_4_ twice a week from postnatal week 10 to week 28, and were dissected 2 days after 28 weeks to examine liver injury. **(B)** Hematoxylin and eosin staining (H&E) staining of liver sections in liver-specific ERBB4 knockout and control mice at week 18 and week 28, respectively. **(C)** After sacrificing, serum ALT levels of liver-specific ERBB4 knockout and control mice were tested (n1=8, n2=8). **(D)** Masson staining of liver sections in liver-specific ERBB4 knockout and control mice at week 18 and week 28, respectively.

To further determine the role of ERBB4 in chronic liver inflammation, we evaluated the effect of ERBB4 in mice models induced by long-term CCl_4_ injection. The experimental design was shown in Figure 5A. Mice were injected with CCl_4_ twice a week from postnatal week 10 to week 28, and were dissected 2 days after 28 weeks to examine liver injury. Liver sections in liver-specific ERBB4 knockout mice (ERBB4^loxp/loxp^;Albcre) and control mice (ERBB4^loxp/loxp^) were performed H&E staining at week 18 and week 28, respectively. Liver-specific ERBB4 knockout mice exhibited severe liver cells injury compared with control mice, especially at week 28 (Figure 5B). Serum ALT levels in liver-specific ERBB4 knockout mice were higher compared with controls (Figure 5C), which was consistent with histological findings. Though liver-specific ERBB4 knockout mice exhibited more severe liver injury, fibrosis was not more severe than controls. Masson staining of liver sections was performed in different groups at week 18 and week 28, respectively. Pseudolobules were found in both groups, but no fibrosis difference was detected (Figure 5D). These results indicated that ERBB4 was protective from liver injury, but not from liver fibrosis.

### Loss of ERBB4 was associated with disrupted lipid metabolism

**Figure 6:**
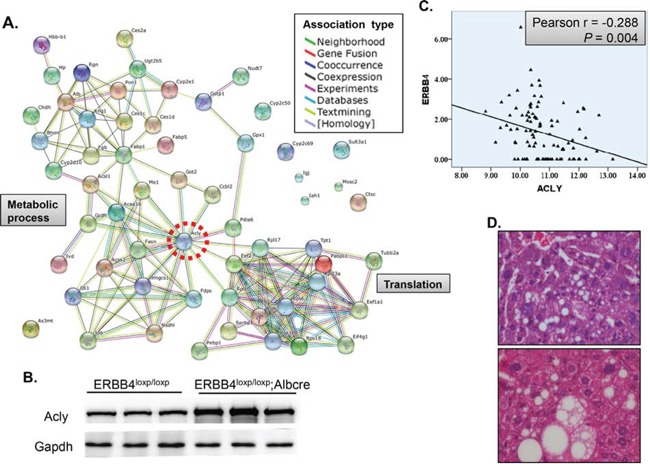
ACLY, an enzyme key for de novo lipogenesis, was negatively associated with ERBB4 **(A)** Protein-protein association of dysregulated proteins after liver-specific knockout of ERBB4. **(B)** Western blot validation in another three pairs of liver-specific knockout and control mice. **(C)** Negative association between ERBB4 and ACLY expression in liver tissues derived from The Cancer Genome Atlas (TCGA) database. **(D)** Lipid accumulation in liver sections of liver-specific ERBB4 knockout mice by hematoxylin and eosin staining (H&E).

Since ERBB4 plays protective role during liver injury, the underlying mechanism is still unclear. We performed quantitative proteomics to identify liver proteomic profile change between a pair of ERBB4 knockout and control mice with ages over 6 months old. Cross validation was applied. We identified 35 increased and 24 decreased proteins between liver-specific knockout and control mice (Supplemental Table 6). The clustering of these dysregulated proteins was enriched in metabolic process and translation, with ACLY located in the center region (Figure 6A). Thus we validated the protein level change in another three pairs of mice, indicating that ACLY was increased in ERBB4 liver-specific knockout mice (Figure 6B). Then we mined the cancer genome atlas (TCGA) database. Interestingly, in 50 pairs of liver cancer and its para-carcinoma tissues, there is significant negative correlation between ERBB4 and ACLY expression, which was consistent with our findings (Figure 6C). As ACLY is a key enzyme for *de novo* lipogenesis, we went through the liver H&E sections, and found lipid accumulation in liver-specific ERBB4 knockout mice with normal feeding (Figure 6D), which provides another clue between ERBB4 and ACLY. However, the molecular mechanisms between the two proteins needs further study.

## DISCUSSION

Here, we identified two genetic variants at 3′UTR of *ERBB4* (rs6147150 and rs1836724) associated with reduced risk for chronic HBV infection. In addition, the variant genotypes are correlated with higher ERBB4 expression. Previously rs6147150 Ins>Del has been found associated with increased HCC risk in a small Chinese population (270 cases and 270 controls) [29]. However, in this study, with relative large population, more systematic SNPs selection and more stable variation detection method, we identified both rs6147150 and rs1836724 associated with decreased risk for chronic HBV infection. In addition, genetic variants associated with chronic HBV infection and HCC has seldom overlapped in the previous GWAS[5, 30–33], indicating different molecular process involved in the two pathogenesis.

In order to confirm the genetic findings, a variety of functional studies were performed. In the HepG2.2.15 cell lines, ERBB4 promoted HBV clearance. In liver section of HBV transgenic mice, we found that both the expression and location of ERBB4 was disrupted, suggesting the impairment of ERBB4 in liver injury. Then, we showed that loss of ERBB4 leads to more severe inflammation both in acute and chronic liver injury mice models, further supporting that ERBB4 is protective in the homeostasis of liver. However, the role of host-immunity in HBV clearance is also vital. Unfortunately, the information for host immune factors in our case-control study is not clear enough, therefore, it is very difficult to evaluate the association between ERBB4 genetic variation and the activation of host-immunity. A more well-designed patients-based study is needed.

Then proteomic quantification showed protein profile change between ERBB4 liver-specific knockout and control mice. ACLY attracted our attention. It is a key enzyme that is involved in *de novo* lipogenesis by catalyzing conversion of cytosolic citrate into acetyl CoA and oxaloacetate[34]. Though the exact role of ACLY in inflammation is not clear, several hints indicate its potential role in inflammation. For example, ACLY mRNA and protein levels markedly and quickly increase in activated macrophages [35]. Interestingly, the mRNA expression between ERBB4 and ACLY was negatively correlated based on human liver tissues from TCGA database. However, these results are very preliminary and merit further investigation.

In summary, genetic association study and functional exploration of ERBB4 uncover its protective effect in liver injury and the 3′UTR genetic variants could be genetic protective markers for chronic HBV infection.

## MATERIALS AND METHODS

### Study subjects

In the case-control study, the subjects' enrollment was described previously [26]. All study subjects were screened for the HBV/HCV markers from two cities in Jiangsu Province (9720 persons from Changzhou and 48422 persons from Zhangjiagang) in 2004 and 2009, respectively. About 865 (8.9%) HBV carriers and 1759 (18.1%) subjects with HBV natural clearance were identified from Changzhou; while 2156 (4.5%) HBV carriers and 7851 (16.2%) subjects with HBV natural clearance were identified from Zhangjiagang. We randomly selected 1344 HBV carriers and 1344 HBV natural clearance subjects from the two cities. This case-control study was approved by the institutional review board of all of the participating institutions.

HBV carriers were positive for both HBV surface antigen (HBsAg) and antibody to hepatitis B core antigen (anti-HBc), negative for hepatitis C virus antibody (anti-HCV). Subjects with HBV natural clearance were negative for HBsAg and anti-HCV, plus positive for both antibody to hepatitis B surface antigen (anti-HBs) and anti-HBc. HBsAg, anti-HBs, anti-HBc and anti-HCV were detected by the enzyme-linked immunosorbent assay (Kehua Bio-engineering Co., Ltd., Shanghai, China) in the serum following the manufacturer's instructions as described previously [26].

### SNPs selection and genotyping

Genetic variants at the regulatory region, including the 5′ and 3′ untranslated region (UTR) of *ERBB4* were selected. The selection flow was shown in Supplemental Table 1. SNPs at 5′ and 3′UTR were predicted whether they may affect transcriptional factors or miRNA binding by online tools including TFBS, miRanda or miRBase. Previously reported SNPs were also included. Finally, rs10182996 at 5′UTR and rs6147150, rs1836724 and rs12467225 at 3′UTR (Figure 1A), were investigated in the case-control study.

Genomic DNA was extracted from a leukocyte pellet by traditional proteinase K digestion, phenol-chloroform extraction and ethanol precipitation. The primers and probes used for detection of variants were presented in Supplemental Table 2. All four variants were genotyped by the TaqMan allelic discrimination assay on a 7900 system (Applied Biosystems, NY, USA). The genotyping was performed without knowing the subjects' case or control status. Two blank (water) controls in each 384-well plate were used for quality control and more than 5% samples were randomly selected and repeated, yielding a 100% concordant.

### Cell culture

The cervical cancer cell line, HeLa, and hepatoma cell line, HepG2.2.15 and QGY-7703 were obtained from Cell Bank at the Chinese Academy of Sciences (Shanghai, China) were cultured in DMEM medium (Gibco, NY, USA) supplemented with 10% fetal bovine serum (Gibco, NY, USA). The cell lines were maintained in a humidified chamber with 5% CO_2_ at 37°C. Transfection was performed by Lipofectamine 2000 (Invitrogen, CA, USA) according to the manufacturer's instructions.

### Plasmids preparation

The fragments (rs6147150 Ins>Del) corresponding to *ERBB4* 3′-UTR region was generated by synthesis and cloned into the pMIR-REPORT vector (Promega, Madison, USA) and the resultant plasmids were designated as pMIR-Ins and pMIR-Del, respectively. All insertions were sequenced to verify the accuracy.

### Transfection and luciferase assays

We seeded 5×10^5^ HeLa, HepG2.2.15 and QGY-7703. The plasmids of pMIR-Ins and pMIR-Del were co-transfected with pRL-SV40, respectively. All transfections were carried out in triplicate. After 48 hours of incubation, cells were collected and analyzed for luciferase activity with the Dual-Luciferase Reporter Assay System (Promega Madison, USA). During transefection, miRNA mimics, inhibitors or controls were added into different groups, respectively. All miRNA mimics, inhibitors and controls were synthesized in RiboBio Co. (Guangzhou, China).

### RNA extraction and quantitative PCR (qRCR) analysis

Total RNA was extracted using TRIzol Reagent (Takara, Dalian, China). First-strand cDNA was synthesized using a reverse transcriptase cDNA synthesis kit (Takara, Dalian, China). For qPCR analysis, aliquots of double-stranded cDNA were amplified using a SYBR Green PCR Kit (Takara, Dalian, China) and 7900 real-time PCR system (Applied Biosystems, NY, USA). The primers used for detection of mRNA of genes related in human and mice were shown in Supplemental Table 3. GAPDH was amplified as an endogenous control. Comparative quantification of target gene was determined using the 2^−ΔΔCT^ method.

### Mouse model of liver injury and liver tumor

Full ERBB4 mutant mice were rescued from embryonic lethality by transgenic ERBB4 expression in the heart (ERBB4^−/−heart^) [27]. ERBB4^−/−heart^ mice were backcrossed to C57BL/6J mice for more than 15 generations. Liver-specific knockout mice (ERBB4^loxp/loxp^; Alb-cre) was generated from ERBB4^loxp/loxp^ crossed with Alb-cre mice. Mouse genotyping was performed by PCR with genomic DNA extracted from the tips of the tails of the mice. Primers used for ERBB4^−/−heart^ mice genotyping were as follows: ERBB4-1:TGT GCG CAG GAA CAG AGA AC; ERBB4-2: CCG CAG GAA GGA GAG GTC; ERBB4-3: CTG CAG GAG ACT AGT GAG AC. Primers used for ERBB4^loxp/loxp^ mice genotyping were as follows: F: AAA TCA TCC TCT TGT GTG CTT TTG TAC; R: CTC GGT ACT GCT GTT CCA GGT CAGA. Primers used for Alb-cre mice genotyping were as follows: F: AAA TCA TCC TCT TGT GTG CTT TTG TAC; R: CTC GGT ACT GCT GTT CCA GGT CAGA. All mice were maintained under a 12/12 h light/dark cycle and received food *ad libitum*. Animal procedures were reviewed and approved by the Animal Care and Use Committee of Soochow University.

In acute carbon tetrachloride (CCl_4_) induced mouse model, CCl_4_ was dilated to 20% concentration by oil. 20% CCl_4_ or pure oil were injected into 2- to 3-month-old male C57BL/6J littermates through intraperitoneal injection (0.25μl/g). Animals were dissected at 12h, 24h, 48h, and 72h after injection to assess liver injury. In long term CCl_4_ -induced mouse model [28], 20% CCl_4_ or pure oil were injected into 2- to 3-month-old male C57BL/6J littermates through intraperitoneal injection (0.25μl/g). The injection was performed twice a week lasted from week 10 to week 28. and the animals were dissected 2 days after 28 weeks to examine histological changes.

lipopolysaccharides (LPS)-induced liver injury model of mice was performed as follows. A solution of LPS (1 mg/ml in PBS; Sigma-Aldrich, MO, USA) was injected into the vena cava at the dosage of 0.5 mg per 25 g body weight of male mice (2- to 3-month-old littermates) and livers were harvested for biochemistry and histology at different time point, including 1, 3, and 13 hr.

### Histology

Liver fixation in paraffin and hematoxylin and eosin (H&E) staining of 4 μm sections were performed using standard protocols. Liver histology was examined by light microscopy in a blinded fashion.

### Serological and biochemical analyses

Venous blood was collected by bleeding of the retro-orbital sinus. Serum was separated after clotting. Alanine aminotransferase (ALT) and aspartate aminotransferase (AST) levels in serum were measured.

### Proteomic quantification

Tissues from ERBB4^loxp/loxp^ (L1 for short) and ERBB4^loxp/loxp^;Albcre (AL1 for short)were homogenized in RIPA lysis buffer (50 mM HEPES containing 150 mM NaCl, 0.1% SDS, 1% Triton X-100, 1% sodium deoxycholate, pH 7.2) with fresh protease and phosphatase inhibitor cocktail (Roche, NJ, USA) on ice for 15 min and centrifuged at 14,500 *g* for 30 min. Protein concentration was measured by BCA protein assay (Pierce, IL, USA). Cold acetone (−80°C) with six time volume of 260 μg protein was added into the tissue lysate and frozen in −80°C overnight. Precipitate was obtained by centrifugation, washed with cold acetone twice, and denatured by 8 mM urea in 50 mM HEPES (pH 8.0). The disulfides in proteins were reduced using 10 mM dithiothreitol (DTT) at room temperature for 1 h, and the resulting free thiols were alkylated with 40 mM iodoacetamide (IAA) for 45 min in dark at room temperature. The excess IAA was quenched using 10 mM DTT. Aliquots of protein were digested using endoproteinase Lys-C (Wako, Japan) at a ratio of 1:100 Lys-C:protein for 3 h. The samples were diluted four times with 50 mM HEPES (pH 8) and further digested overnight with trypsin (Promega, WI, USA) at a ratio of 1:50 trypsin:protein. Full digestion of proteins was validated by silver staining. Digested peptides from L1 and AL1 mice were labeled with TMT-126 and TMT-127 labeling reagents, respectively (Thermo Scientific, IL, USA). The labeling reaction was proceeded for 1 hr at room temperature, and then the sample was treated with hydroxylamine (Sigma, MO, USA). Then the labeled peptides from L1 and AL1 mice were mixed in a 1:1 ratio and desalted using solid-phase C18 extraction cartridges (Waters, MA, USA) and dried in a vacuum centrifuge. Peptides were resuspended in 20 mL of 0.1% formic acid and analyzed in HPLC-MS/MS. After higher energy collision dissociation (HCD)-induced cleavage in MS/MS, the TMT tags are cleaved to generate two different masses for relative quantification of the peptides from two samples. The peptides were identified by database search with Proteome Discoverer 1.3 (Thermo Scientific, IL, USA). False positive rates (1%) were controlled using the target-decoy database search with the concatenated reversed database. Peptides less than 7 amino acids in length were excluded. TMT reporter 126/127 values in the MS/MS scans were used to quantify the relative abundance of each protein. The reciprocal labeling of two samples was also conducted.

### Statistical analysis

The Student's t-test and χ^2^ test were used to detect differences of demographic characteristics, genotype frequencies of the SNP between the cases and controls for continuous variables and categorical variables, respectively. Associations between the genotypes and risk of chronic HBV infection were estimated by computing odds ratios (ORs) and their 95% confidence intervals (CIs) from logistic regression analyses. The adjusted ORs were from multivariate logistic regression with the adjustment for age, gender, drinking and smoking status. The Chi-square-based *Q* test was applied to test the heterogeneity of associations between subgroups. Gene expression difference between two groups were calculated by the Student's t-test. All tests were two-sided. All of the statistical analyses were performed with R software (version 2.13.0; The R Foundation for Statistical Computing).

## SUPPLEMENTARY TABLES


