# Supplementary material for: Genetic variants in ERBB4 is associated with chronic hepatitis B virus infection
Source: Oncotarget. 2015 Dec 18;7(4):4981–92. doi: 10.18632/oncotarget.6650 (PMC4826259; doi:10.18632/oncotarget.6650)
Supplement: Supplementary file 1 [file oncotarget-07-4981-s001.pdf]

SUPPLEMENTARY TABLES

Supplementary Table S1: SNPs selection flow

rs. No.	Location	TFBS	miRanda	miRBase	Reported	Selected
rs10182996	5’UTR	Y	--	--	--	Y
rs10932374	3’UTR	--	--	--	--	--
rs12467225	3’UTR	--	Y	Y	--	Y
rs12471583	3’UTR	--	Y	--	--	--
rs13431425	3’UTR	--	--	--	--	--
rs1595066	3’UTR	--	Y	--	--	--
rs16846013	3’UTR	--	--	--	--	--
rs1836734	3’UTR	--	--	--	--	--
rs1971801	3’UTR	--	--	--	--	--
rs1971802	3’UTR	--	--	--	--	--
rs2887992	3’UTR	--	--	--	--	--
rs3791691	3’UTR	--	--	--	--	--
rs3791696	3’UTR	--	--	--	--	--
rs3791700	3’UTR	--	--	--	--	--
rs4673613	3’UTR	--	--	--	--	--
rs4673614	3’UTR	--	--	--	--	--
rs6749560	3’UTR	--	--	--	--	--
rs10048757	3’UTR	--	--	--	--	--
rs11895168	3’UTR	--	Y	--	--	--
rs12992904	3’UTR	--	--	--	--	--
rs13014117	3’UTR	--	--	--	--	--
rs13025037	3’UTR	--	--	--	--	--
rs1583483	3’UTR	--	--	--	--	--
rs1595062	3’UTR	--	--	--	--	--
rs1595065	3’UTR	--	Y	--	--	--
rs1816533	3’UTR	--	--	--	--	--
rs1821656	3’UTR	--	--	--	--	--
rs1836724	3’UTR	--	Y	Y	--	Y
rs1972820	3’UTR	--	Y	--	--	--
rs3791692	3’UTR	--	--	--	--	--
rs4672612	3’UTR	--	Y	--	--	--
rs4673610	3’UTR	--	--	--	--	--
rs6710253	3’UTR	--	--	--	--	--
rs7592118	3’UTR	--	--	--	--	--
rs934607	3’UTR	--	--	--	--	--
rs6147150	3’UTR	--	--	--	Y	Y

Supplementary Table S2: The primers and probes used for detection of genetic variations within the promoter and 3' untranslated region (UTR) of *ERBB4*

Genetic variations	The primers	Probes
**rs10182996**	F: TGTCCTCACTCTGCACATTCATG	allele T: FAM-TCCCTAATCTGGAGGTC-MGB
**T>C**	R: TAAAAGGCAAGGGATATGTACATTG	allele C: HEX-TCCCTAATCCGGAGGT-MGB
**rs6147150**	F: TTACCACTTTCTTTAGGCAATCATTTAAC	allele Ins, HEX-CATACCTGAAATAGGAT-MGB
**Ins>Del**	R:TGAGACAAATTAGTATTAAAATTACACGGAGTA	allele Del: FAM-ATACCTGCCCAAAAT-MGB
**rs1836724**	F: CACCACAAAAGGTGTTAGACTACCA	allele T: FAM-AGCTCTTGGTTTTC-MGB
**T>C**	R: GCGTTGTTTTTCATGCTATTTTAAGA	allele C: HEX-CAGCTCCTGGTTTT-MGB
**rs12467225**	F: AAACTTGTATTTACATCCTTCCCTG	allele T: FAM-AGCTTCACACAGTC-MGB
**C>T**	R: GAAAGACATGGCATGAAGATTGT	allele C: HEX- AGCCTCACACAGTC-MGB

Supplementary Table S3: The primers used for detection of gene expression by real-time PCR in this study

Gene Symbol	Species	Primers
ERBB4	human	F: GCC TCT GGA GAA TTT ACG CAT
		R: GGG TTC CGA ACA ATA TCT TGC C
GAPDH	human	F: ACA ACT TTG GTA TCG TGG AAG G
		R: GCC ATC ACG CCA CAG TTT C
ERBB4	mouse	F: CCT TCC TGC GGT CTA TCC GA
		R: CCA AAG TTG CCA TCT TTC CTG TA
GAPDH	mouse	F: TGC CCA GAA CAT CAT CCC TG
		R: TCA GAT CCA CGA CGG ACA CA
IFN-γ	mouse	F: GCT CTG AGA CAA TGA ACG CTA CA
		R: TTT CTT CCA CAT CTA TGC CAC TT
TNF-a	mouse	F: CAG GCG GTG CCT ATG TCT C
		R: CGA TCA CCC CGA AGT TCA GTA G
RANTES	mouse	F: TTT GCC TAC CTC TCC CTC G
		R: CGA CTG CAA GAT TGG AGC ACT
IL-6	mouse	F: CTG CAA GAG ACT TCC ATC CAG TT
		R: GAA GTA GGG AAG GCC GTG G
IL-10	mouse	F: AGC CTT ATC GGA AAT GAT CCA GT
		R: GGC CTT GTA GAC ACC TTG GT
IL -12	mouse	F: GTG GAC CAA ACA ATC TGA CCT G
		R: AAC ACG GAC TAT GAA CCT GGA

Supplementary Table S4: All SNPs identified by imputation analyses at ERBB4 and their associations with chronic HBV infection (*P* < 0.05)

SNPs	Locations	Minor Allele	OR[a]	*P*[a]
rs7565960	212242434	A	0.8586	0.03818
rs13003941	212243703	T	0.8592	0.04063
rs72947669	212244033	C	0.8592	0.04063
rs10932374	212244403	A	0.8595	0.04076
rs12467225	212244657	T	0.8592	0.04063
rs12471583	212244718	C	0.8592	0.04063
rs1836724	212244952	G	0.747	0.02488
rs12475523	212245284	A	0.8545	0.03386
chr2:212246253	212246253	T	0.8545	0.03386
rs3791682	212249706	C	0.8545	0.03386
chr2:212250835	212250835	C	0.8568	0.03705
rs34975993	212257285	A	0.8513	0.01593
rs12990779	212263271	A	1.141	0.049
rs16846049	212263838	C	1.143	0.04646
rs12992515	212267373	C	1.142	0.04865
chr2:212291941:D	212291941	D	0.8642	0.03419
rs6710946	212295875	C	0.8138	0.01599
rs6435622	212296094	C	0.8138	0.01599
rs13414946	212296470	T	0.8099	0.01401
chr2:212297514:D	212297514	D	0.823	0.02254
chr2:212298148	212298148	C	0.8327	0.02789
rs13384614	212298311	A	0.8139	0.01401
rs6435623	212300372	G	0.7906	0.006019
rs6726567	212335333	G	1.156	0.04192
rs6743937	212375963	C	0.7525	0.01487
rs6730119	212519028	G	1.166	0.03217
rs10190533	212520406	A	1.163	0.03674
chr2:212521051:I	212521051	I	1.163	0.03674
rs34621071	212522651	G	1.189	0.01922
rs10932391	212523114	C	1.163	0.03674
rs4422103	212526145	A	1.172	0.02728
rs71422749	212526751	A	1.161	0.03688
rs13019783	212538415	G	0.7836	0.0486
chr2:212577763	212577763	C	0.7817	0.04765
rs72946553	212580143	G	0.7817	0.04765
rs12470126	212867522	A	0.7048	0.003594

(Continued)

SNPs	Locations	Minor Allele	OR[a]	*P*[a]
chr2:212867715	212867715	T	0.7048	0.003594
rs1402714	212868114	C	0.7048	0.003594
rs1879637	212868624	C	0.7048	0.003594
rs72613739	212869056	A	0.7048	0.003594
rs960824	212871592	G	0.7048	0.003594
rs6733992	212872058	C	0.7048	0.003594
rs6734102	212872139	C	0.7048	0.003594
rs6734634	212872426	G	0.7048	0.003594
chr2:212873590	212873590	G	0.7048	0.003594
rs12464843	212874828	G	0.7048	0.003594
rs12477159	212874848	C	0.7048	0.003594
rs10169217	212875346	A	0.6992	0.002948
rs7595560	212875540	C	0.7738	0.0404
chr2:212875625	212875625	A	0.7672	0.03455
rs12476274	212877782	A	0.7672	0.03455
chr2:212878653	212878653	G	0.7672	0.03455
rs59081924	212879401	T	0.7617	0.0298
chr2:212881929	212881929	C	0.7617	0.0298
rs1473636	212882190	T	0.7617	0.0298
chr2:212882485	212882485	G	0.7617	0.0298
rs1402716	212882926	G	0.7617	0.0298
rs1473637	212883345	T	0.7617	0.0298
rs1473638	212883383	G	0.7617	0.0298
rs12466520	212885278	C	0.7617	0.0298
rs1521537	212885510	T	0.7617	0.0298
chr2:212886000	212886000	C	0.7388	0.01882
chr2:212886345	212886345	C	0.7617	0.0298
rs6435684	212886373	G	0.7617	0.0298
chr2:212886423	212886423	C	0.7617	0.0298
rs6715981	212886857	G	0.7617	0.0298
chr2:212886873:D	212886873	D	0.7617	0.0298
chr2:212887351	212887351	A	0.7617	0.0298
chr2:212887394	212887394	A	0.7617	0.0298
chr2:212887517	212887517	G	0.7742	0.0376
chr2:212888711	212888711	T	0.7742	0.0376
rs7574462	212888988	C	0.7742	0.0376
chr2:212889491	212889491	T	0.7742	0.0376

(*Continued*)

SNPs	Locations	Minor Allele	OR[a]	P[a]
chr2:212889514	212889514	G	0.7617	0.0298
rs61193005	212890848	A	0.7617	0.0298
rs2139939	212891276	T	0.7617	0.0298
rs12478950	212892250	C	0.7617	0.0298
chr2:212892543	212892543	A	0.7617	0.0298
rs1402718	212894077	G	0.7617	0.0298
chr2:212894879	212894879	C	0.7742	0.0376
chr2:212895638	212895638	A	0.7742	0.0376
chr2:212895646	212895646	A	0.7742	0.0376
rs73087372	212895680	T	0.7742	0.0376
rs1546717	212902339	A	0.7742	0.0376
chr2:212903130	212903130	C	0.7617	0.0298
rs60885616	212904323	A	0.7617	0.0298
chr2:212904901:I	212904901	I	0.7617	0.0298
rs12468427	212906168	A	0.7742	0.0376
rs16847568	212907324	G	0.7617	0.0298
rs12470737	212908043	A	0.7617	0.0298
rs16847575	212908414	A	0.7742	0.0376
rs7582903	212908837	A	0.7742	0.0376
rs12476163	212909135	G	0.7742	0.0376
rs10192934	212910060	G	0.7742	0.0376
chr2:212910644	212910644	G	0.7742	0.0376
rs10445808	212913367	C	0.7633	0.03044
rs16847580	212914356	G	0.7742	0.0376
rs1464443	212914726	C	0.7617	0.0298
chr2:212915498:I	212915498	I	0.7742	0.0376
rs12473708	212915640	A	0.7617	0.0298
rs6712126	212916157	A	0.7617	0.0298
rs939645	212916758	G	0.7742	0.0376
chr2:212917505	212917505	T	0.7742	0.0376
rs2012211	212919796	T	0.7742	0.0376
rs4672633	212920334	T	0.7617	0.0298
rs12474504	212921683	G	0.7815	0.04419
chr2:212921708	212921708	A	0.7732	0.03432
rs12474510	212921862	C	0.7732	0.03432
chr2:212922181	212922181	T	0.7732	0.03432
chr2:212922724:D	212922724	D	0.7732	0.03432

(*Continued*)

SNPs	Locations	Minor Allele	OR[a]	*P*[a]
rs10180520	212924118	A	0.761	0.0272
rs10170406	212928179	C	0.7732	0.03432
rs4672634	212928499	C	0.7732	0.03432
rs4672636	212929014	G	0.7732	0.03432
rs10197270	212929113	A	0.7732	0.03432
chr2:212930568	212930568	A	0.7584	0.02629
rs2139940	212934247	T	0.7584	0.02629
chr2:212934625	212934625	C	0.752	0.02228
chr2:212934704:D	212934704	R	0.752	0.02228
chr2:212935459:D	212935459	R	0.752	0.02228
rs12373751	212936891	T	0.752	0.02228
rs9678219	212937649	T	0.752	0.02228
chr2:212939165	212939165	T	0.7708	0.03329
rs17343912	212939754	A	0.7708	0.03329
rs17343932	212941020	G	0.7708	0.03329
rs17415843	212941258	C	0.7708	0.03329
rs4672637	212943207	A	0.763	0.02786
rs56993927	212944667	C	0.763	0.02786
chr2:212945757	212945757	C	0.762	0.02753
rs10497958	212945959	C	0.762	0.02753
rs10210742	212947028	T	0.762	0.02753
rs17344051	212947356	G	0.762	0.02753
rs17344065	212947759	G	0.762	0.02753
rs4672638	212957507	G	0.7727	0.0466
rs2371436	212957816	C	0.7749	0.04768
rs1357125	212958253	C	0.7727	0.0466
rs1521550	212958457	T	0.7738	0.02501
chr2:212959713:I	212959713	I	0.7662	0.03394
chr2:212961017:D	212961017	R	0.7662	0.03394
rs2371438	212965531	G	0.7672	0.03434
rs13017857	212966007	A	0.7662	0.03394
chr2:212966812:D	212966812	D	0.7672	0.03434
rs34306607	212967059	G	0.7727	0.03973
chr2:213151321:I	213151321	R	0.6058	0.01071
rs11686852	213151472	G	0.6855	0.02488
chr2:213151492:I	213151492	R	0.645	0.02714
rs7575288	213153280	A	0.645	0.02714

(*Continued*)

SNPs	Locations	Minor Allele	OR[a]	*P*[a]
rs10205688	213155360	G	0.6555	0.03385
rs10205716	213155534	C	0.6555	0.03385
rs9288451	213156090	A	0.6555	0.03385
rs17346713	213156498	G	0.6555	0.03385
rs2371481	213156797	G	0.6555	0.03385
rs72945692	213158191	T	0.6555	0.03385
chr2:213158432	213158432	T	0.6555	0.03385
chr2:213158433	213158433	T	0.6555	0.03385
rs72945695	213158529	C	0.6555	0.03385
rs17418640	213158820	G	0.6555	0.03385
rs72945697	213158862	T	0.6555	0.03385
rs4672644	213163621	C	0.6663	0.042
rs10932432	213164193	T	0.6663	0.042
rs17418814	213164792	A	0.6663	0.042
rs11679625	213165830	G	0.6617	0.04044
rs2371480	213166049	T	0.6663	0.042
rs1505364	213185208	C	0.6627	0.01217
rs10164675	213187034	T	0.6299	0.02069
rs12694275	213188134	A	0.646	0.03118

NOTE: [a]Logistic regression analysis adjusted for age, gender and the first principal component.

Supplementary Table S5: Stratified analyses on association between rs6147150 and risk of chronic HBV infection

Variables	HBV Carriers II/ID/DD	HBV Clearances II/ID/DD	OR(95%CI)	$P_{heterogeneity}$
Age				
≤53	405/255/38	359/280/58	**0.78 (0.66-0.93)**	0.288
>53	367/230/43	349/233/54	0.89 (0.75-1.06)	
Gender				
Male	651/415/67	598/428/91	**0.85 (0.75-0.97)**	0.463
Female	121/70/14	110/85/21	0.75 (0.55-1.02)	
Smoking status				
Ever	439/283/45	409/303/66	**0.82 (0.70-0.97)**	0.776
Never	333/202/36	299/21046	0.85 (0.71-1.03)	
Drinking status				
Ever	340/225/34	291/220/56	**0.78 (0.65-0.94)**	0.331
Never	432/260/47	417/293/56	0.88 (0.75-1.03)	

NOTE: Multivariate logistic regression analyses adjusted for age, sex, smoking status and drinking status in dominant genetic model (excluded the stratified factor in each stratum).

Supplementary Table S6: Quantitative proteomic analyses of liver proteins in liver-specific ERBB4 knockout and control mice

Name	Description	L1:AL1	AL1:L1
Hmgcs1	Hydroxymethylglutaryl-CoA synthase, cytoplasmic	0.366	2.748
Idi1	Isopentenyl-diphosphate Delta-isomerase 1	0.444	2.717
Acly	ATP-citrate synthase	0.511	2.273
Gstp1	Glutathione S-transferase P 1	0.484	2.250
Fdps	Farnesyl pyrophosphate synthase	0.575	2.180
Pdia6	Protein disulfide-isomerase A6	0.632	1.995
Lss	Lanosterol synthase	0.331	1.993
Fasn	Fatty acid synthase	0.562	1.917
Acss2	Acetyl-coenzyme A synthetase, cytoplasmic	0.536	1.631
Eif4g1	Eukaryotic translation initiation factor 4 gamma 1 (Fragment)	0.555	1.581
Me1	Malic enzyme	0.690	1.477
Nudt7	Isoform 5 of Peroxisomal coenzyme A diphosphatase NUDT7	0.716	1.475
Nsdhl	Sterol-4-alpha-carboxylate 3-dehydrogenase, decarboxylating	0.611	1.421
Bhmt	Betaine--homocysteine S-methyltransferase 1	0.697	1.417
Rpl10	60S ribosomal protein L10 (Fragment)	0.762	1.398
Rpl23a	60S ribosomal protein L23a	0.785	1.372
Rpl17	60S ribosomal protein L17	0.678	1.361
Eef2	Elongation factor 2	0.740	1.361
Serbp1	Isoform 4 of Plasminogen activator inhibitor 1 RNA-binding protein	0.710	1.333
Eef1a1	Elongation factor 1-alpha 1	0.787	1.330
Ivd	Isovaleryl-CoA dehydrogenase, mitochondrial	0.765	1.326
Fabp5	Fatty acid-binding protein, epidermal	0.767	1.300

(*Continued*)

Name	Description	L1:AL1	AL1:L1
Fgb	Fibrinogen beta chain	0.762	1.277
Tubb2a	Tubulin beta-2A chain	0.768	1.273
Rps6	40S ribosomal protein S6	0.737	1.263
Rps18	40S ribosomal protein S18	0.792	1.256
Tpt1	Translationally-controlled tumor protein	0.724	1.255
Cyp2d10	Cytochrome P450 2D10	0.793	1.244
Pabpc1	Polyadenylate-binding protein 1	0.740	1.242
Rpl6	60S ribosomal protein L6	0.765	1.230
Hp	Haptoglobin	0.767	1.223
Rps16	40S ribosomal protein S16	0.781	1.215
As3mt	Arsenite methyltransferase	0.719	1.207
Acsl1	Very long-chain acyl-CoA synthetase	0.783	1.206
Ugt2b5	UDP glucuronosyltransferase 2 family, polypeptide B5	0.790	1.201
Ces2a	Pyrethroid hydrolase Ces2a	1.233	0.806
Ccbl2	cysteine conjugate-beta lyase 2	1.224	0.791
Kng1	Isoform LMW of Kininogen-1	1.227	0.784
Alb	Serum albumin	1.463	0.767
Fabp1	Fatty acid-binding protein, liver	1.610	0.758
Chdh	Choline dehydrogenase, mitochondrial	1.278	0.743
Rgn	Regucalcin	1.714	0.736
Gcdh	Glutaryl-CoA dehydrogenase, mitochondrial	1.367	0.733
Gpx1	Glutathione peroxidase 1	1.345	0.732
Iah1	Isoamyl acetate-hydrolyzing esterase 1 homolog	1.245	0.728
Cyp2c50	Isoform 2 of Cytochrome P450 2C50	1.278	0.719
Ces1c	Carboxylesterase 1C	1.440	0.708
Igj	Immunoglobulin J chain (Fragment)	1.498	0.666

(*Continued*)

Name	Description	L1:AL1	AL1:L1
Pon1	Serum paraoxonase/ arylesterase 1	1.458	0.664
Ces1d	Carboxylesterase 1D	1.566	0.657
Hbb-b1	Hemoglobin subunit beta-1	1.451	0.657
Ctsc	cathepsin C	1.409	0.654
Pebp1	Phosphatidylethanolamine-binding protein 1	1.727	0.610
Sult3a1	Amine sulfotransferase	1.669	0.596
Acaa1b	acetyl-Coenzyme A acyltransferase 1B	1.309	0.578
Cyp2c69	Protein Cyp2c69	1.434	0.478
Cyp2e1	Cytochrome P450 2E1	1.228	0.000
Got2	Kynurenine--oxoglutarate transaminase 1	1.236	0.000
Marc2	Mitochondrial amidoxime reducing component 2	1.248	0.000
